# Transcriptomic analysis of starch accumulation patterns in different glutinous sorghum seeds

**DOI:** 10.1038/s41598-022-15394-1

**Published:** 2022-07-01

**Authors:** Fulai Ke, Kuangye Zhang, Zhihua Li, Jiaxu Wang, Fei Zhang, Han Wu, Zhipeng Zhang, Feng Lu, Yanqiu Wang, Youhou Duan, Zhiqiang Liu, Jianqiu Zou, Kai Zhu

**Affiliations:** Sorghum Research Institute, Liaoning Academy of Agricultural Sciences, Shenyang, 110161 Liaoning Province People’s Republic of China

**Keywords:** Agricultural genetics, Plant physiology

## Abstract

Sorghum is a high-quality raw material for brewing white wine, and the starch content in seeds has a large impact on brewing quality. Transcriptomic data obtained from a glutinous variety (Liaonian3) and a non-glutinous variety (Liaoza10) at 3, 18, and 30 days after pollination were analyzed to identify genes associated with starch accumulation. The amylopectin content was significantly higher in Liaonian3 compared to Liaoza10, but the amylose content and total starch content were lower. There were 6634 differentially expressed genes found in Liaoza10 between 3 and 18 d after pollination, and 779 differentially expressed genes between 18 and 30 d after pollination. In Liaonian3, there were 6768 differentially expressed genes between 3 and 18 d after pollination, and 7630 differentially expressed genes between 18 and 30 d after pollination. Genes were grouped by expression profiles over the three time points and the profiles were analyzed for enrichment of gene ontology terms and biochemical pathways. Profile 1 (decreasing expression from 3 to 30 d) for Liaoza10 was enriched in ribosomes, metabolic pathways, and carbon metabolic pathways. Profile 0 (decreasing expression from 3 to 18 d and consistent expression from 18 to 30 d) was enriched in pathways related to sugar or starch metabolism. Although the starch accumulation rate in Liaonian3 and Liaoza10 showed a profile of increasing and then decreasing, the expression of genes related to starch synthesis gradually decreased with time since pollination, demonstrating the complexity of starch synthesis. According to orthologous gene alignment and expression analysis, 19 genes such as *entrzID_8068390* and *entrzID_8066807* were found to be the key genes for starch synthesis and glutinous and non-glutinous differentiation in sorghum grains.

## Introduction

*Sorghum bicolor* (L.) Moench (sorghum) seeds can be divided generally into glutinous and japonica (non-glutinous) types. In wine-making, yield is closely related to the amylopectin content in the seeds, and glutinous sorghum, in addition to being rich in amylopectin grains, has characteristics such as higher starch digestibility and ethanol conversion^1^. Some studies have shown that glutinous sorghum with higher amylopectin content is more suitable than japonica sorghum as a raw material for wine-making, e.g., the famous traditional Chinese wines Moutai and Wuliangye^2^.

Starch synthesis in the endosperm is a complex metabolic process. Granule-bound starch synthase (GBSS) is a key enzyme in starch synthesis and is closely related to the synthesis of amylose. GBSS activity affects the apparent structure of the endosperm and changes the content and ratio of amylose and amylopectin^3^. Glutinous genes are widely present in cereal crops, such as rice and wheat^4,5^. Pedersen et al. (2005) first classified glutinous traits in sorghum into wx^a^ and wx^b^ types based on reduced GBSS enzyme activity and clarified the dominant-recessive relationship between wild-type Wx, wx^a^, and wx^b^^6^. Hamblin et al. (2007) analyzed variations of the gene encoding the GBSS protein and identified a non-synonymous single mutation site associated with the glutinous trait. Differences in this site result in either a glutamine or histidine at position 268 in the GBSS protein, which affects the glutinous trait in sorghum^7^. McIntyre et al. (2008) found nine InDel loci and 24 SNP loci through comparative analysis of the GBSS gene sequence of a glutinous and a japonica variety, BTxARG1 and QL39, respectively. They identified two SNPs that result in non-synonymous substitutions in the GBSS protein. Through map-based cloning, they determined that the *wx* gene is located on chromosome 10^8^. Sattler et al. (2009) analyzed the allelic mutations *wx*^*a*^ and *wx*^*b*^ of two waxy genes in sorghum and found that *wx*^*a*^ contains a large insertion in the third exon of the gene encoding GBSS, leading to a decrease in GBSS protein content. Their findings in *wx*^*b*^ were consistent with the results of the Hamblin study mentioned above^9^. In addition, researchers identified a novel waxy allele, wx^c^, was found in a Taiwanese landrace.This allele consists of a + 1G to C mutation in the 5′ splice site at the intron 10–exon 11 boundary. A DNA marker specific for wx^c^ was produced to distinguish the wx^c^ allele from other alleles, allowing the identification of heterozygous non-waxy plants^10^.Although some previous studies related to glutinousness of sorghum seeds have been conducted, the regulation of sorghum starch synthesis at the RNA level is currently unclear.

To fill this knowledge gap, we here analyzed the gene expression and gene regulatory pathways/networks of starch synthesis in glutinous and japonica sorghum. This was accomplished through transcriptomic analysis of two common and widely-used sorghum varieties, Liaonian3 and Liaoza10. Our results clarify the patterns of starch accumulation in different types of sorghum and provide a theoretical basis for the selection and cultivation of high amylopectin sorghum varieties.

## Results

### Starch accumulation rate of Liaonian3 and Liaoza10

**Figure 1:**
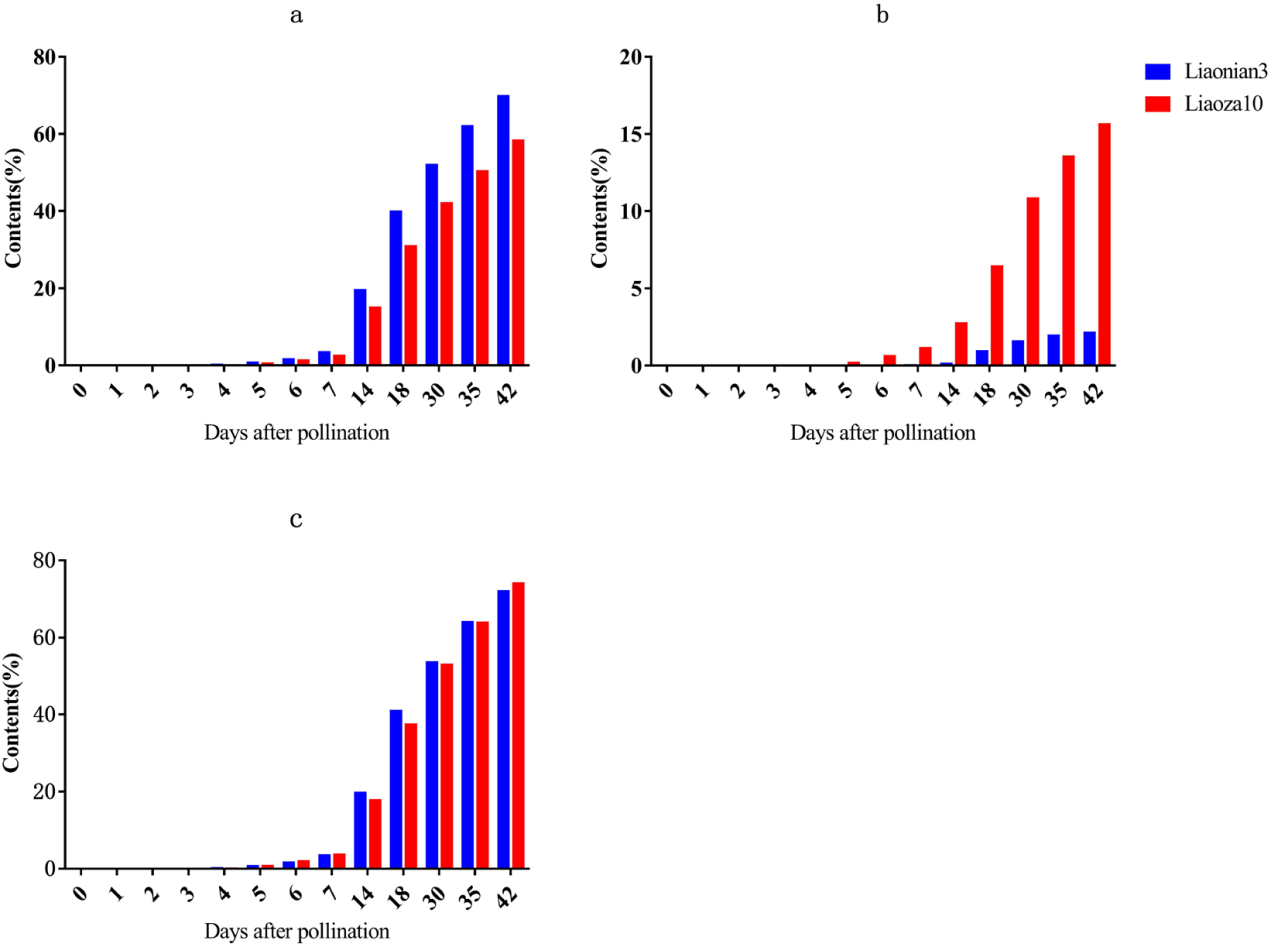
Changes of Starch Content in Liaonian3 and Liaoza 10. Note: (**a**) amylopectin; (**b**) amylose; (**c**) total starch.

The amylopectin, amylose, and total starch content of sorghum seeds were measured at 0, 1, 2, 3, 5, 6, 7, 14, 18, 30, 35, and 42 days after pollination (Fig. 1). Both Liaonian3 and Liaoza10 started to accumulate amylopectin and amylose from the third day after pollination; the accumulation rate gradually increased, peaked at 18 d, then gradually decreased. After 42 d, the amylopectin contents of Liaonian3 and Liaoza10 were 70.09% and 58.59%, respectively, whereas levels of amylose were much higher in Liaoza10 (15.7%) than in Liaonian3 (2.2%). The total starch contents of Liaonian3 and Liaoza10 were 72.29% and 74.29%, respectively, a difference that was not statistically significant (*t* = − 1.405, *p* = 0.173).

### Transcriptome data quality control analysis

RNA-seq was performed on six samples of Liaoza10 at 3 d (J3), 18 d (J18), and 30 d (J30) after pollination, and on Liaonian3 at 3 d (N3), 18 d (N18), and 30 d (N30) after pollination. We obtained 39.6, 40.4, 38.4, 38.9, 41.9, and 40.2 megabase pairs (Mbp) of raw data for these samples, respectively. After filtering the six samples for quality, they yielded 39.5, 40.3, 38.3, 38.8, 41.7, and 40.1 Mb of clean data, respectively. The validity of the six samples’ data ratios were all above 99%. The filtered RNA-seq data of all six samples had Q20 ratios above 98%, Q30 ratios between 94.83% and 95.19%, and GC contents conformed to normal values between 51.5% and 53.5%. In summary, the RNA-seq data for all six samples were of good quality, confirming the suitability for subsequent analyses (Table 1).

**Table 1:** Squencing data quality statistics.

Sample	RawData(bp)	CleanData(bp)	AF_Q20(%)	AF_Q30(%)	AF_GC(%)
J3	39,596,497	39,483,589(99.71%)	98.38	95.11	51.53
J18	40,431,825	40,320,059(99.72%)	98.42	95.19	52.37
J30	38,419,073	38,297,395(99.68%)	98.28	94.83	52.79
N3	38,939,086	38,836,315(99.74%)	98.4	95.1	51.52
N18	41,861,037	41,747,149(99.73%)	98.31	94.9	52.93
N30	40,205,917	40,074,515(99.67%)	98.31	94.93	53.51

### Analysis of gene expression quantity and abundance

**Figure 2:**
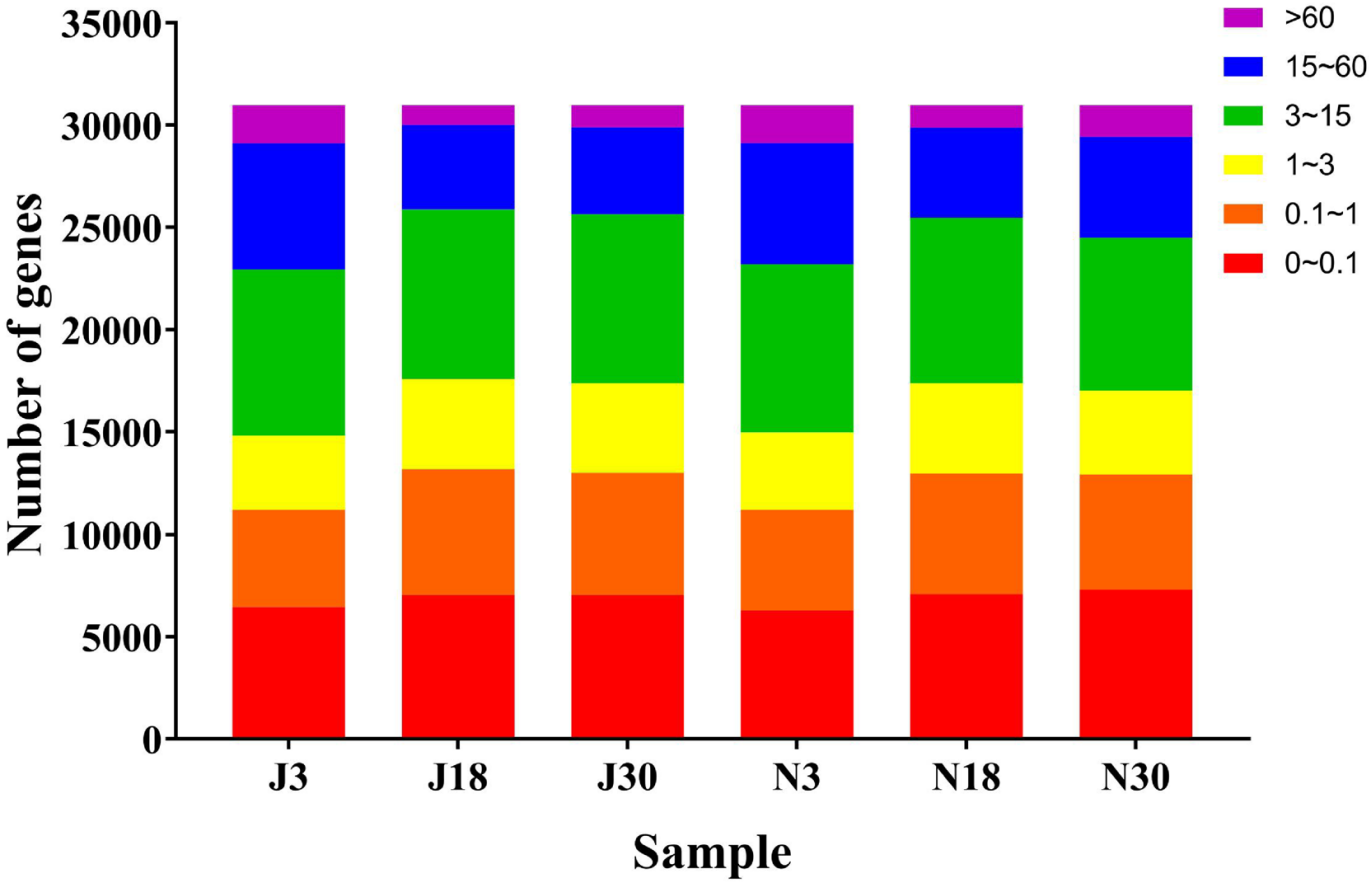
The number of genes in different expression levels.

A total of 185,832 genes were identified among the six samples, and the distribution of FPKM values was analyzed (Fig. 2). The most common FPKM values were between 3 and 15, with over 24% of genes from all samples falling in this range. The least abundant in all samples were genes with FPKM values above 60; the smallest number of genes in any FPKM category in any sample was 960 genes with FPKM > 60 in Liaonian3 at 18 d. Most of the genes were expressed at relatively high levels, with over 76% of genes from all samples having FPKM values > 0.1.

### Screening for differentially expressed genes (DEGs)

**Figure 3:**
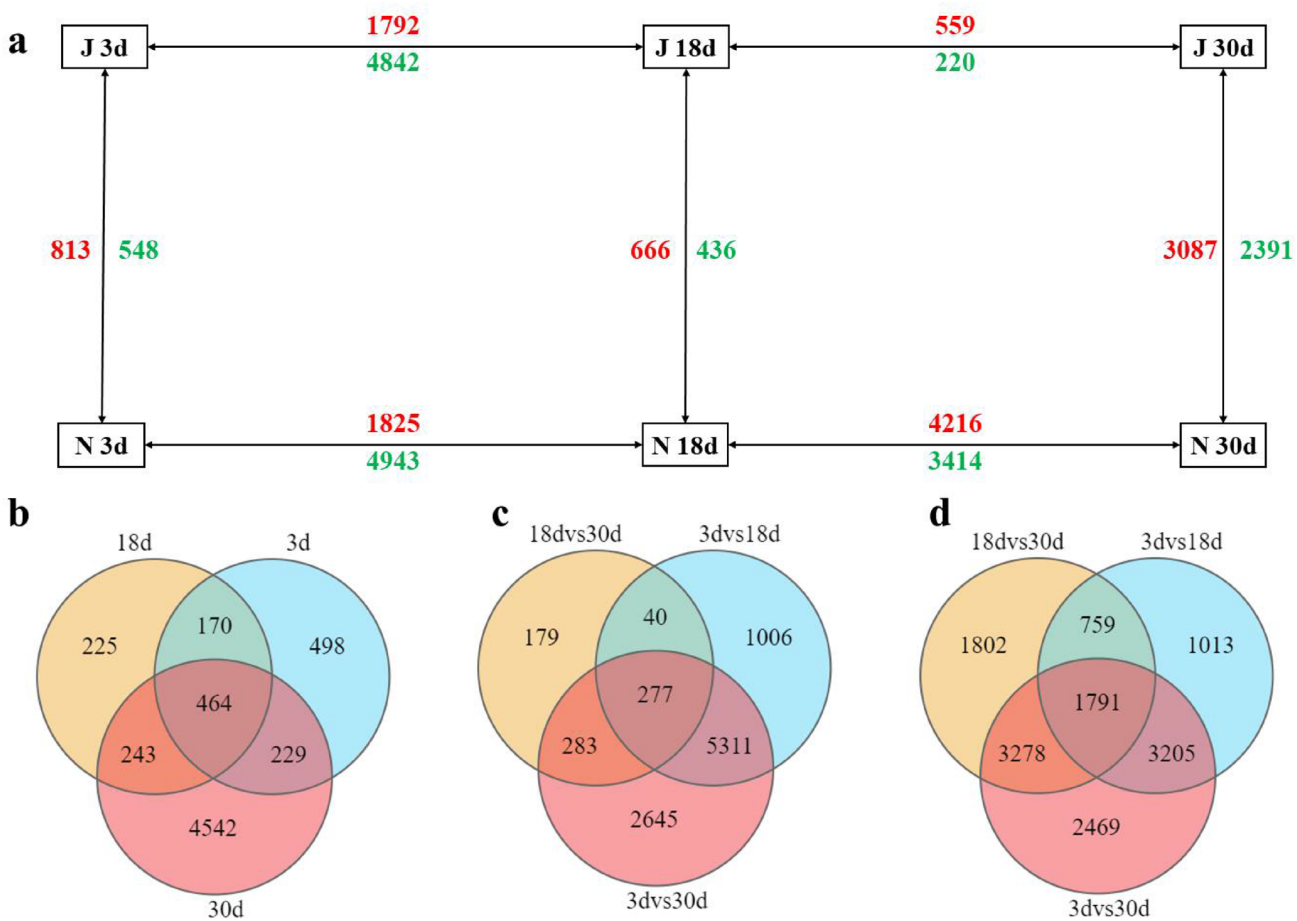
Diagram of differential gene expression among different samples. Note: (**a**) the number of differential genes among the six samples, red represents up-regulated expression, green represents down-regulated expression; (**b**) Venn diagram of differential genes between Liaonian3 and Liaoza10; (**c**) Venn diagram of differential gene in different periods for Liaoza10; (**d**) Venn diagram of differential gene in different periods for Liaonian3.

In Liaoza10, there were 6634 DEGs between the 3 d and 18 d samples, of which 1792 were up-regulated and 4842 were down-regulated. There were only 779 DEGs between 18 and 30 d, of which 559 were up-regulated and 220 were down-regulated. In Liaonian3, there were 6768 DEGs between 3 and 18 d after pollination, including 1825 up-regulated and 4943 down-regulated genes. There were 7630 DEGs between 18 and 30 d, including 4216 up-regulated and 3414 down-regulated genes (Fig. 3).

At 3 days after pollination, there were 1361 DEGs between Liaoza10 and Liaonian3, of which 813 were up-regulated and 548 were down-regulated. At 18 d after pollination, there were 1102 DEGs between the two varieties, with 666 up-regulated and 436 down-regulated. After 30 days of pollination, there were many DEGs between the two varieties, totaling 5478, including 3087 genes up-regulated and 2391 genes down-regulated. Among the genes differentially expressed between the two varieties, there were 464 genes that were significantly different at all three time points (Fig. 3).

### Profile analysis

#### Post-pollination gene expression profile analysis of Liaonian3

**Figure 4:**
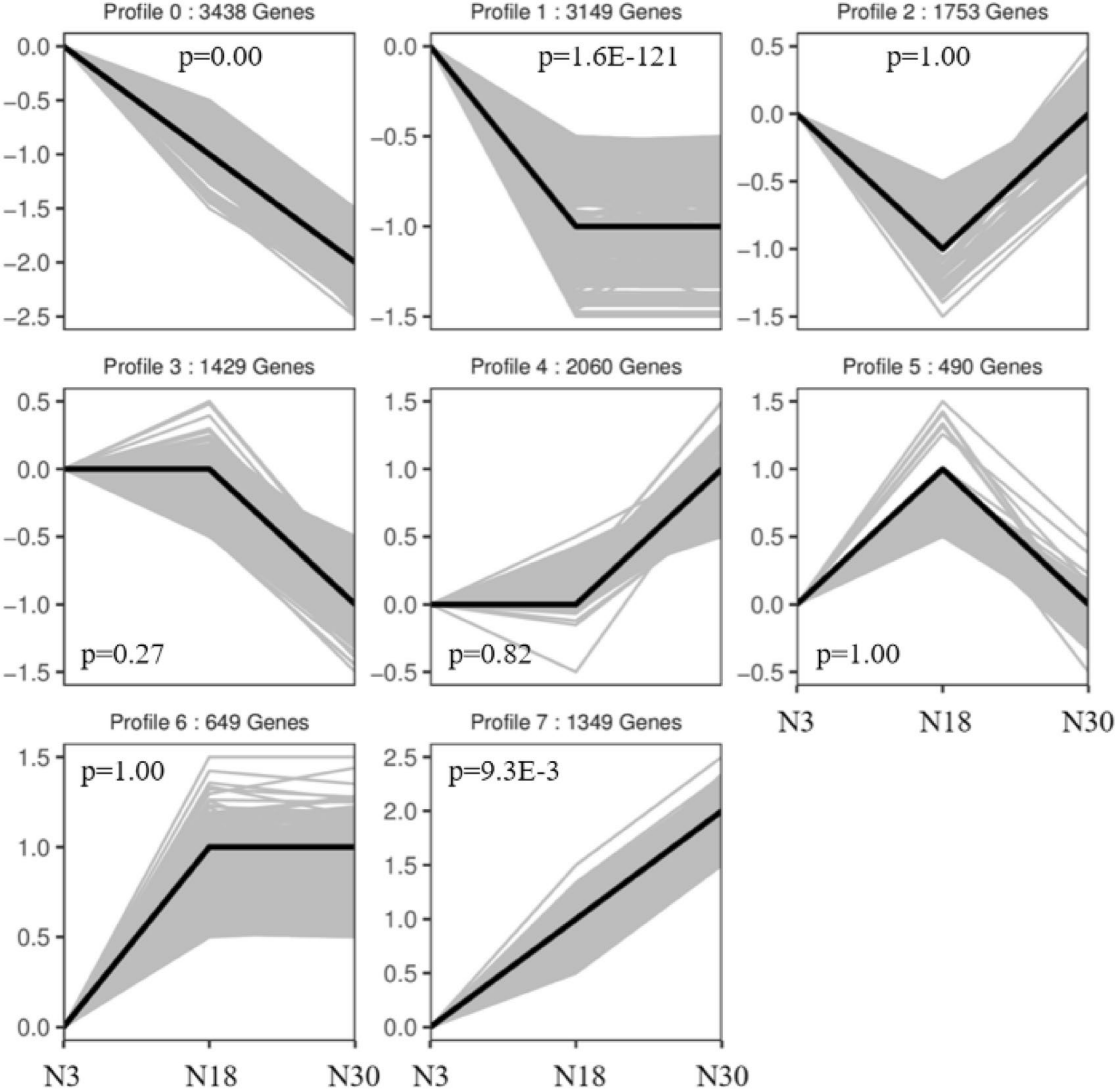
Gene expression profile information of Liaonian3.

Analysis of gene expression at 3 d, 18 d, and 30 d after pollination of the glutinous Liaonian3 yielded eight gene expression patterns, referred to as profiles (Fig. 4). Expression of genes in Profile 0 gradually decreased between 3 and 30 d; Profile 1 showed a decrease between 3 and 18 d, then remained constant between 18 and 30 d; Profile 2 decreased between 3 and 18 d, then increased; Profile 3 was approximately constant between 3 and 18 d then decreased at 30 d; Profile 4 was approximately constant between 3 and 18 d then increased; Profile 5 was approximately increasing between 3 and 18 d then decreasing; Profile 6 was approximately increasing between 3 and 18 d, then constant; and Profile 7 gradually increased between 3 and 30 d. The highest number of genes in this dataset (3438) followed Profile 0, followed by Profile 1 (3149), and Profile 5 had the lowest number of genes (490). Gene expression profile analysis was followed by annotation of individual genes using GO and KEGG, then subsequent GO term and pathway enrichment analysis for each profile.

**Figure 5:**
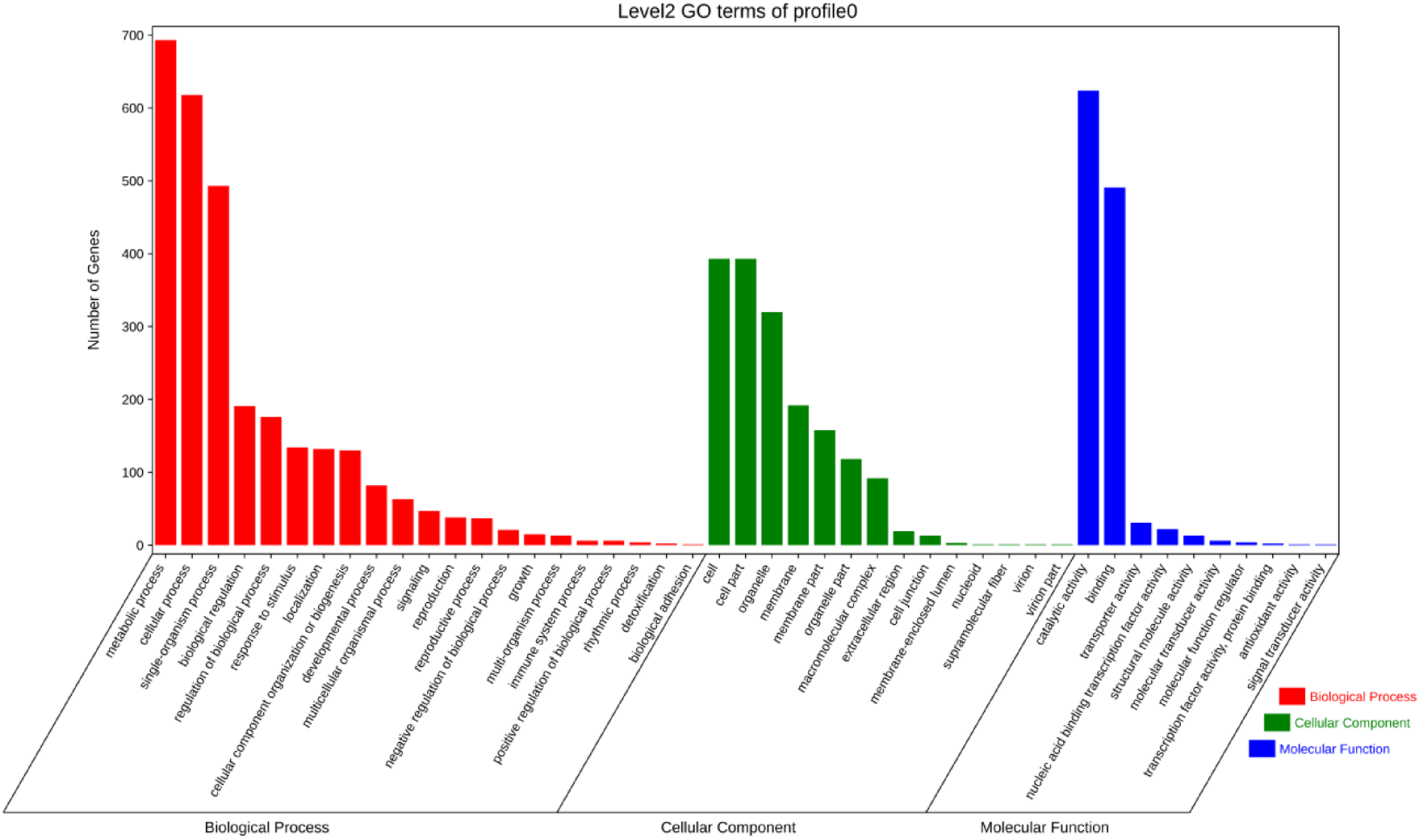
Profile 0 gene GO enrichment classification histogram of Liaonian3.

The smallest *p*-value was found for Profile 0 in Liaonian3. The top three biological process GO terms for genes in this profile were metabolic process (693), cellular process (618), and single organ process (493) (Fig. 5); the top three cellular component GO terms were cellular composition (393), intracellular(367) and organelles (320); and the top three molecular function GO terms were catalytic activity (624), binding (491), and transport activity (31).

**Figure 6:**
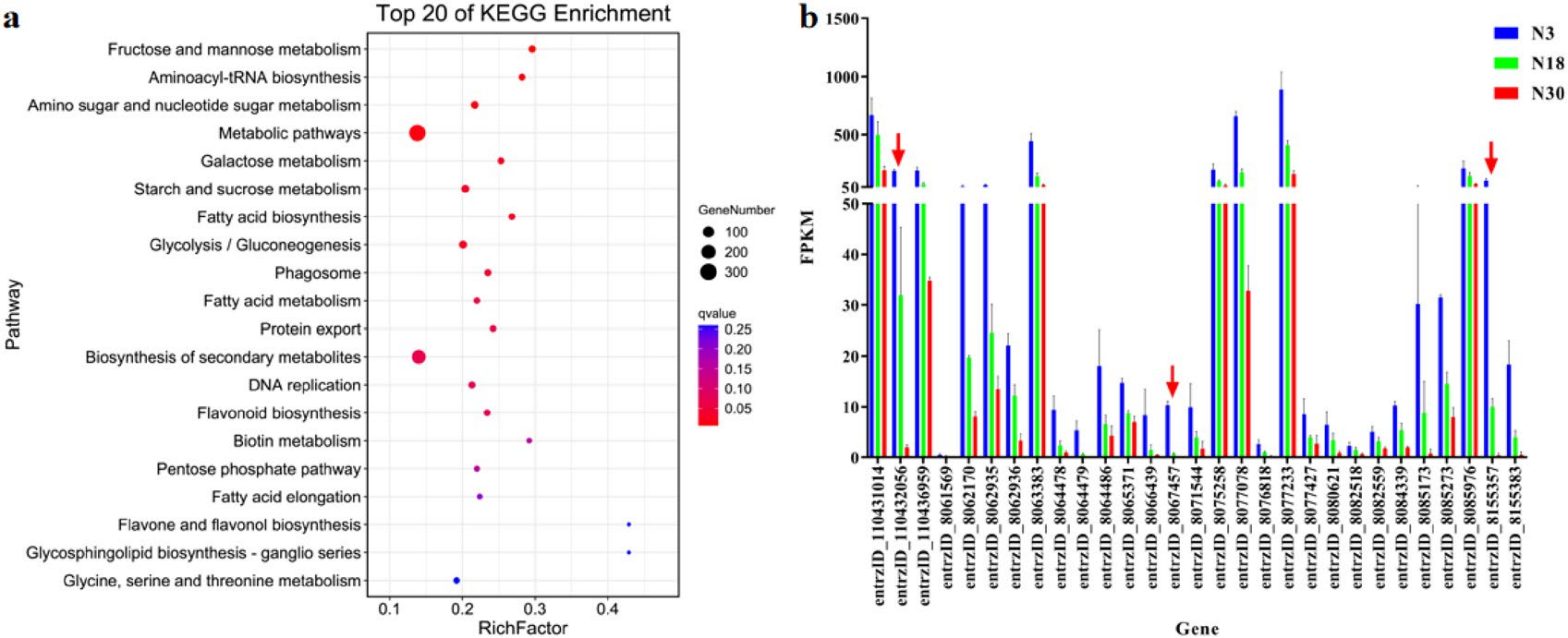
Profile 0 KO enrichment bubble chart and some gene expression levels of Liaonian3. Note: a, profile 0 KEGG analysis of Liaonian3; b, changes in gene expression of starch and sucrose metabolism pathways.

KEGG analysis revealed that Liaonian3 Profile 0 was mainly associated with sugar-related metabolic or synthetic pathways, such as fructose and mannose metabolism, aminosaccharide and ribose metabolism, galactose metabolism, starch and fat metabolic pathways, and glycolysis/glycoisomerization (Fig. 6a). The gene expression pattern described by Profile 0 thus shows that sugar metabolism was most active in the seeds starting at 3 d after pollination, then gradually decreased. There were 29 genes involved in starch and fat metabolism pathways, and the expression of all these genes conformed to a gradual decline. The three genes showing the greatest decline were entrzID_8155357, entrzID_8067457, and entrzID_110432056, which had decline rates of 99.6%, 99.5%, and 98.9%, respectively (Fig. 6b).

#### Post-pollination gene expression profile analysis of Liaoza10

**Figure 7:**
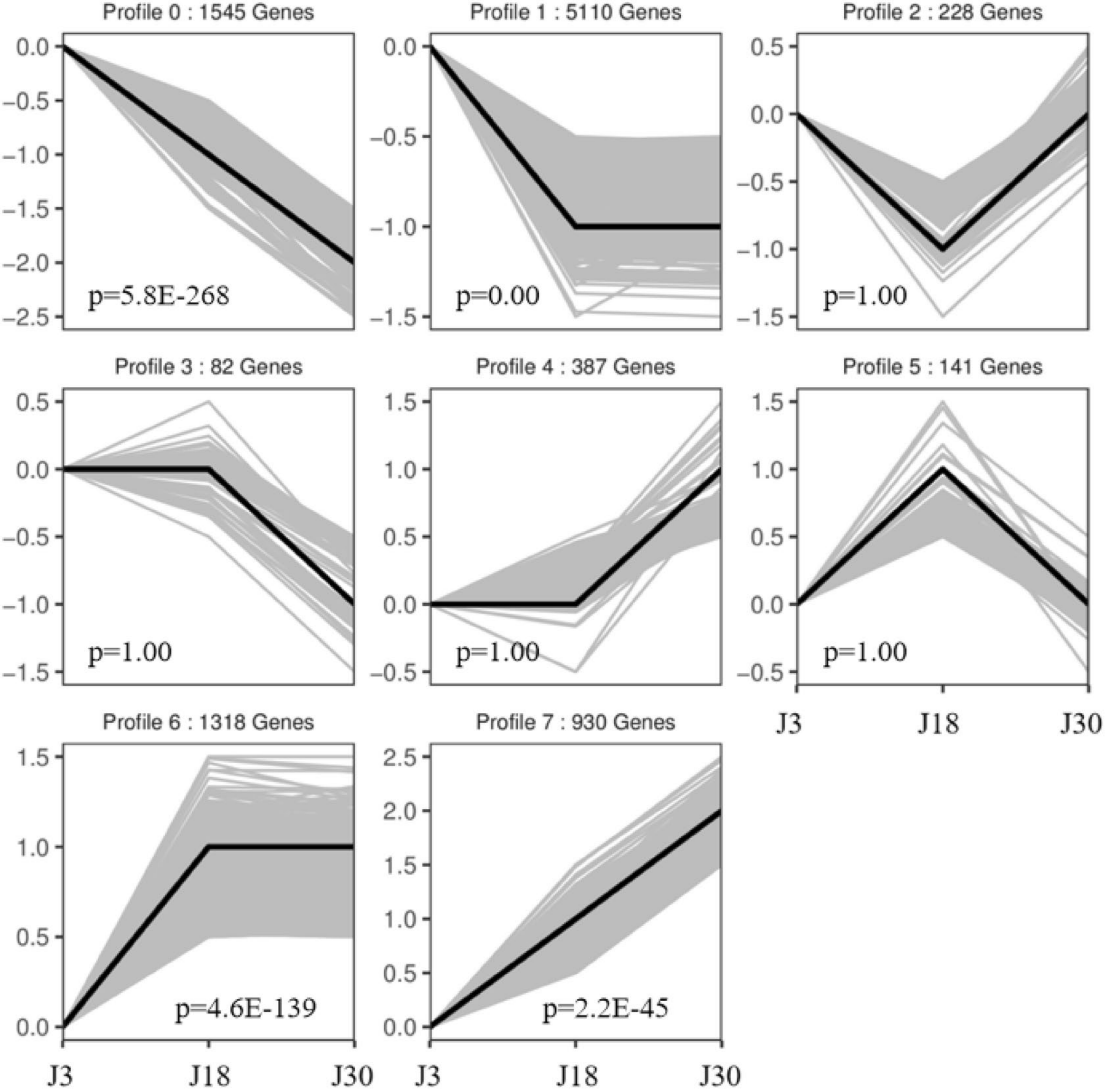
Gene expression profile information of Liaoza10.

Analysis of gene expression in the seeds of japonica sorghum variety Liaoza10 at 3 d, 18 d, and 30 d after pollination yielded eight profiles. Profile 1 described the expression pattern of the most genes (5110) and Profile 3 the fewest genes (82). Profile 1 had the smallest *q*-value and contained genes whose expression decreased from 3 to 18 d then remained constant (Fig. 7).

**Figure 8:**
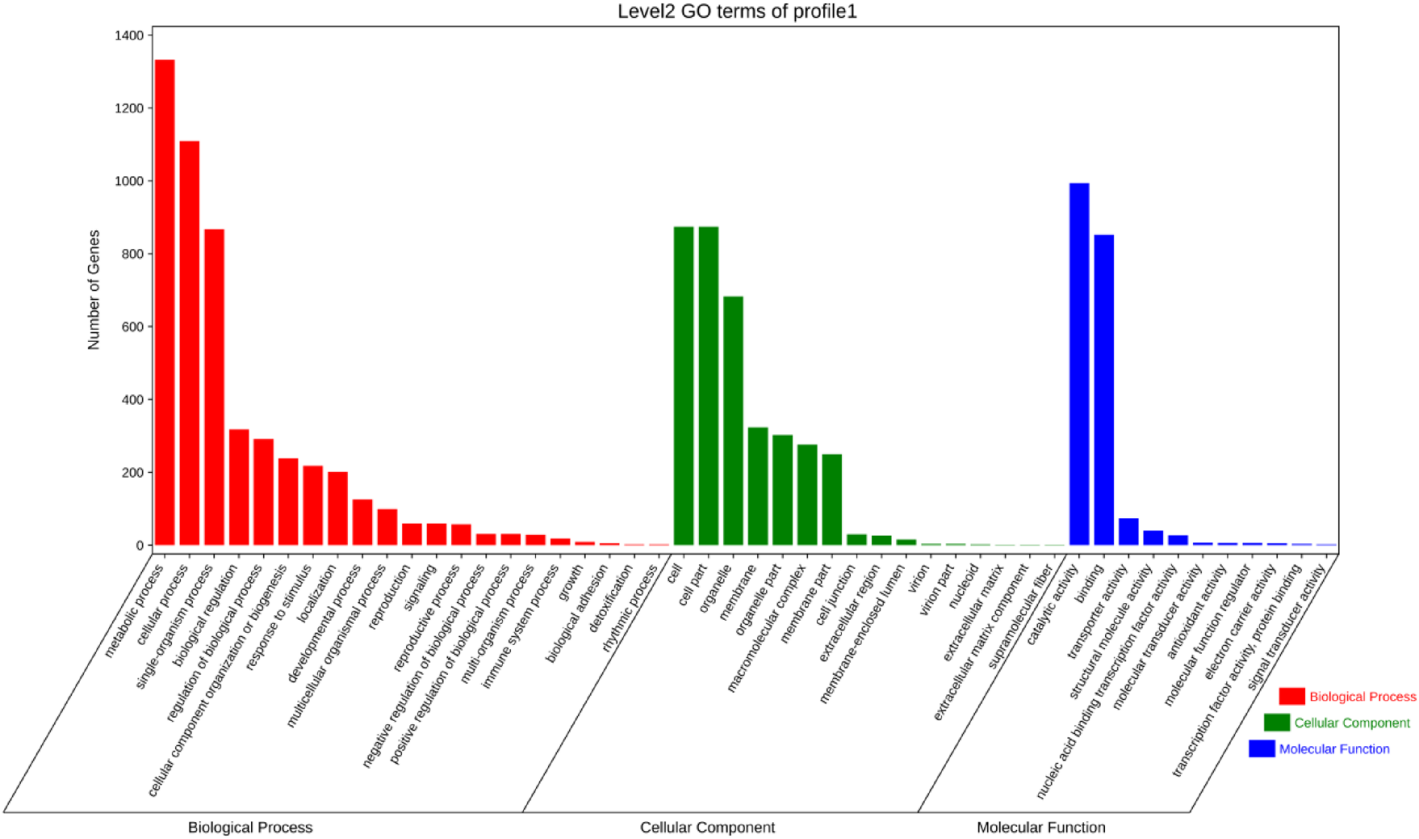
Profile 0 gene GO enrichment classification histogram of Liaoza10.

Enrichment analysis was performed to identify GO terms and KEGG pathways over-represented in Profile 1 of Liaoza10 (Fig. 8). Significantly enriched GO biological processes, cellular components, and molecular functions were similar to those in Profile 1 for Liaonian3. The top three biological process GO terms were metabolic processes (1332), cellular processes (1109), and single organ processes (867); the top three cellular component GO terms were cellular components (874), organelles (683) and intracellular organelle(677); the top three molecular function GO terms were catalytic activity (994), binding (852), and transport activity (74).

**Figure 9:**
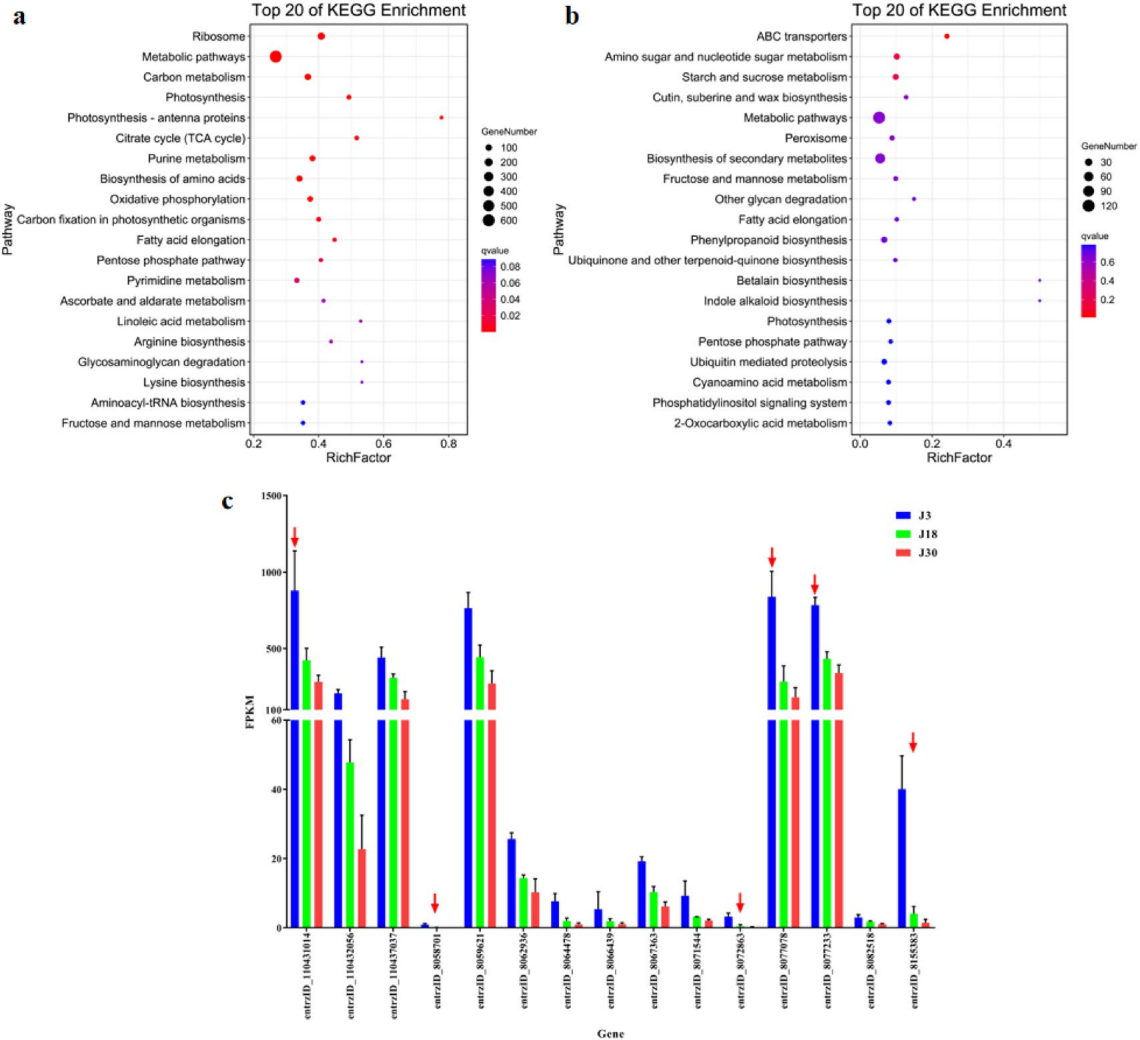
Profile 1 and profile 0 KO enrichment bubble charts and some gene expression levels of Liaoza10. Note: (**a**) profile 1 KEGG analysis of liaoza10; (**b**) profile 0 KEGG analysis of liaoza10; c,changes in gene expression of starch and sucrose metabolism pathways of profile 0.

Pathway analysis was performed for Profile 1 with KEGG annotation, and the top 20 pathways with the smallest *q*-values primarily involved ribosomes, metabolic pathways, and carbon metabolic pathways; no pathways related to sugar metabolism were significantly enriched (Fig. 9a). However, there was a relatively high enrichment of pathways related to sugar or starch metabolism in Profile 0. In Profile 0, the pathway with the lowest *q*-value was the ABC-type transcription factor, followed by the amino- and ribose-metabolism pathway, then the starch and fat metabolism pathway (Fig. 9b). Similar to the results seen in Liaonian3, gene expression in Profile 0 also decreased with time after pollination (Fig. 9c). Analysis of the 15 genes in the starch and fat metabolic pathways in Profile 0 revealed that the three genes with the highest initial expression were entrzID_110431014, entrzID_8077078, and entrzID_8077233*.* The three genes with the greatest rate of decline were entrzID_8058701, entrzID _8155383, and entrzID_8072863, with decrease ratios of 99.0%, 96.2% and 96.1%, respectively.

### Expression of genes related to starch synthesis

**Figure 10:**
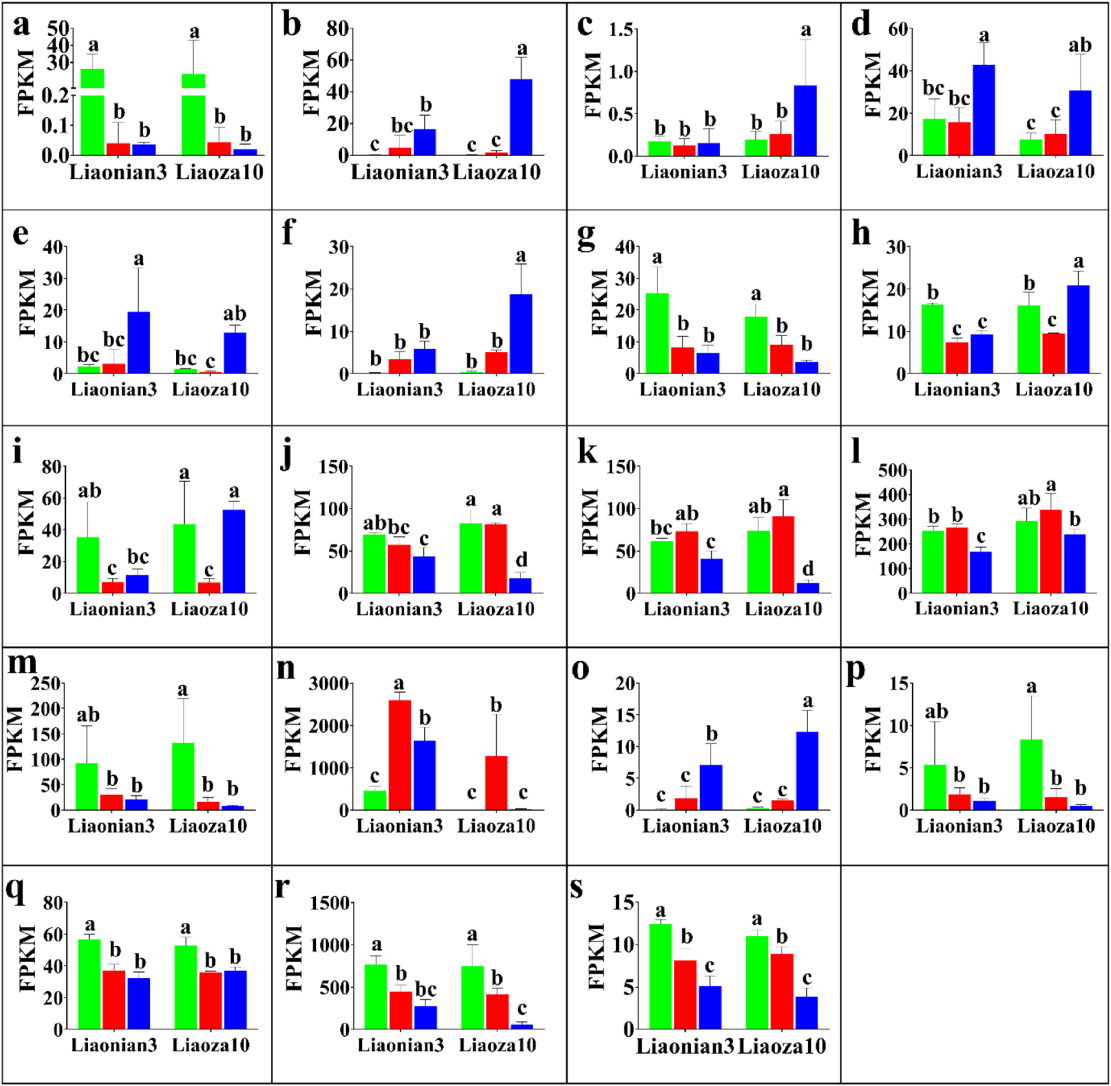
Expression pattern analysis of starch-related gene. Note: (**a**) *entrzID_8075199*; (**b**) *entrzID_110436912*; (**c**) *entrzID_8065370*; (**d**) *entrzID_8056206*; (**e**) *entrzID_8078919*; (**f**) *entrzID_8083539*; (**g**) *entrzID_8069792*; (**h**) *entrzID_8072062*; (**i**) *entrzID_8066807*; (**j**) *entrzID_8085013*; (**k**) *entrzID_8081295*; (**l**) *entrzID_110430978*; (**m**) *entrzID_8057926*; (**n**) *entrzID_8068390*; (**o**) *entrzID_8068151*; (**p**) *entrzID_8066439*; (**q**) *entrzID_8059673*; (**r**) *entrzID_8059621*; (**s**) *entrzID_8056518*.

Because *wx* gene (*entrzID_8068390*) in sorghum was closely associated with starch content and composition in previous studies, we invoked the expression level of *entrzID_8068390* from the RNA-Seq results which were shown in Fig. 10. It can be seen that at 3 days after pollination, there was no significant difference in the expression levels of Liaonian3 and Liaoza10, but from 18 days after pollination onwards, the expression level of *entrzID_8068390* in Liaonian3 (2597.8) was twice as high as that of Liaoza10 (1284.4), and at 30 days after pollination, the expression levels of this gene in Liaonian3 and liaoza10 were 1641.8 and 16.4, respectively, which showed the expression level of this gene in Liaonian3 was significantly higher than that in Liaoza10 (about 99.8-fold). From the above results, it can be seen that *entrzID_8068390* can regulate the sorghum starch content by the level of expression.

In addition to the above *entryzID_8068390* gene, 19 orthologous of starch synthesis-related genes in rice(including *entryzID_8068390*) were queried through NCBI in order to explore other genes related to starch synthesis in sorghum, and the orthologous relationship of these genes in rice were shown in the Appendix 1. The expression levels of each gene in Liaonian3 and Liaoza10 are shown in Fig. 10. After analysis of variance, it was found that the expression levels of *entrzID_110436912*, *entrzID_8065370*, *entrzID_8083539*, *entrzID_8072062*, *entrzID_8066807*, *entrzID_8085013*, *entrzID_8081295*, *entrzID_110430978*, *entrzID_8068390*, and *entrzID_8068151* were significantly different. Among them, *entrzID_8068390* is the homologous of *wx* and *RAG2* in rice, and *entrzID_110436912*, *entrzID_8065370*, *entrzID_8083539* and *entrzID_8072062* are the homologous of *RAmy1A* and *RAmy3D* in rice. E*ntrzID_8066807* and *entrzID_8085013* are orthologous of *OsSSIIIa* and *OsSSIIIb*, *entrzID_8081295* and entrzID_110430978 are orthologous of *OsSSIIa*, and *entrzID_8068151* is orthologous of *RSR1*. At 3 days after pollination, these ten genes were not significantly different in both Liaonian3 and Liaoza10 materials. Seven of these genes showed expression differences at 30 days after pollination in Liaonian3 and Liaoza10, and they were *entrzID_110436912*, *entrzID_8065370*, *entrzID_8083539*, *entrzID_8072062*, *entrzID_8066807*, *entrzID_8081295* and *entrzID_8068151*, among which, only *entrzID_8081295* was significantly more expressed in Liaonian3 than Liaoza10 at 30 days after pollination, while the expression of the other six genes were significantly lower in Liaonian3 than in Liaoza10 at 30 days after pollination. The expression of *entrzID_8085013* and *entrzID_110430978* were significantly higher in Liaonian3 than in Liaoza10 at 18 days after pollination, and at 30 days after pollination, both genes showed a decreasing profile in two materials compared to 18 days after pollination. At 30 days after pollination, the expression of *entrzID_8085013* was significantly lower in Liaoza10 than in Liangnian3 , while the expression of *entrzID_110430978* gene was significantly higher in Liaoza10 than in Liangnian3. Based on the above results, it can be found that all the above ten genes are closely related to starch content and can affect amylopectin content, but it cannot be excluded that the other nine genes were also related to the differentiation of glutinous and non-glutinous, and based on the expression pattern of the above genes, it can be seen that the differentiation of glutinous and non-glutinous should occur after 3 days after pollination in different materials.

## Discussion

In previous studies, it has been found that starch accumulation in sorghum grain follows a sigmoid curve, and genotype-based differences in starch content are not statistically significant in early stages of growth. Significant differences mainly appear in the middle and late stages, and the maximum accumulation rate has appeared to occur in the 21st to 28th day after flowering^11^. We here investigated the starch accumulation patterns of glutinous (Liaonian3) and japonica (Liaoza10) sorghum varieties after pollination, and found that both varieties showed an initial increase in starch accumulation rate followed by a decrease; starch content over time formed a sigmoid curve, meaning these results are generally consistent with those in previous studies.

Starch is a major component of sorghum grain, and starch biosynthesis and the ratio of amylose and amylopectin are closely related to grain yield, milling ratio, palatability, and brewing quality. Previous studies have examined the genetic basis of sorghum grain starch content and found that it is a typical quantitative trait that is easily influenced by the environment. In recent years, researchers have studied the activity of various enzymes during starch accumulation in sorghum seeds. Ke et al. (2020) found that bound starch synthase (GBSS) activity showed a significant positive correlation with the rate of amylose accumulation in japonica and semi-japonica sorghum, but not in glutinous sorghum. It was hypothesized that the regulatory effect of GBSS on amylose was influenced by the substrate concentration, or that the higher activity of starch branching enzyme (SBE) and starch debranching enzyme (DBE) in glutinous sorghum grains inhibited the effect of GBSS; this would ultimately result in the inhibition of amylose regulation^12^. In this study, the seeds of Liaonian3 and Liaoza10 were sampled at 3 d, 18 d, and 30 d after pollination. Transcriptomic data from these seeds indicated that in glutinous and japonica sorghum, genes in the starch synthesis and various sugar metabolic pathways were most active at the end of pollination (3 d) and gradually decreased thereafter. However, starch biosynthesis involves several enzymes, e.g., soluble starch synthase (SSS), GBSS, sucrose synthase (SS), adenosine diphosphate glucose pyrophosphorylase (ADPGPase), and uridine diphosphate glucose pyrophosphorylase (UDPGPase). Starch synthesis is relatively susceptible to environmental influences^13^, and the reason for the decrease in starch synthesis rate at mid-grazing is currently unclear.

In rice, the National Rice Data Center (www.ricedata.cn) have identified 17 genes or QTL that can affect starch content or quality in the grain, of which 11 have been successfully cloned^13–23^. In this study, orthologous of these genes in sorghum were identified by homologous search and comparison. Among these orthologous, except for the *Wx* gene, which has also been shown to regulate amylose content in sorghum, ten other genes were found to have significantly different expression levels in Liaonian3 and Liaoza10 materials by comparing the expression levels of these genes in the two materials. These genes have highly similar sequences to their orthologous in rice, for example, the sequence similarity between the two genes *entrzID_8066807* in sorghum and *OsSSIIIa* in rice is about 79.01% (Appendix 2, cited from the ensembl website), and the two genes share the same structural domain (cl33462). It is assumed that *entrzID_8066807* in sorghum has a similar function to *OsSSIIIa* in rice, and it is well understood that *OsSSIIIa* in rice affects the structure of amylopectin, amylose content and physicochemical properties of starch granule in rice seeds^20^, and the absence and mutation of the gene encoding IIIa will lead to changes in starch-related traits of the grain, which affect the synthesis of resistant starch together with the Waxya allele^21^. Genes such as *OsCBP20*^24^, *RSR1*^14^, can all affect the amylose content in zygotes, with *RSR1* and *OsSSIIIa* also affecting the structure of amylopectin^14,20^. In this study, *entryzID_8066807* was not significantly different in both Liaonian3 and Liaoza10 materials at 3 days after pollination and at 18 days after pollination, and the difference in expression of *entryzID_8066807* in Liaonian3 and Liaoza10 only appeared at 30 days after pollination. Therefore, it is hypothesized that *entrzID_8066807* is a key gene for amylose synthesis in sorghum and affects amylose content mainly in the late stage of seed formation. Among these orthologous in this study, two genes, other than the *wx* gene (*entrzID_8068390*), were expressed at higher levels in both materials. Gene *entrzID_8059621*, which was not significantly different in expression levels at any of the three periods in the two materials, presumably because it is associated with starch synthesis in sorghum but does not affect the differentiation of glutinous and non-glutinous. The other gene is *entrzID_110430978*, which is orthologous to *OsSSI* gene in rice, which affects the structure of amylopectin in the seeds, and mainly the pasting temperature of rice^15^. But when *OsSSI* is deficient, other starch synthesis isozymes can partially compensate for the function of *OsSSI*, while the result of the compensation obviously changed the branch chain length of amylopectin, which showed that the composition of amylopectin was collaboratively completed by isomers *SSI*, *SSIIa*, *SSIIIa*^15,22^^.^ In this study, there was no significant difference in the expression level of this gene in the two materials at 3 days after pollination, and the expression of Liaonian3 was significantly higher than that of Liaoza10 at 18 days after pollination, and in Liaoza10 at 30 days after pollination, the expression was significantly higher than that of Liangnian3. Based on the above, it is presumed that *entrzID_110430978* can regulate the structure of amylopectin in sorghum, and it starts to function at the middle of seed formation, and it still need further validation that other orthologus are also more likely to be closely related to sorghum grain starch glutinous and non-glutinous differentiation.

Sorghum grain starch synthesis is a very complex biological process that is regulated by a variety of enzymes, hormones, and environmental factors^11,12^. In this study, we used RNA-seq to conduct a preliminary study of the grain starch synthesis pathway in glutinous and japonica sorghum varieties. The data show that although the rate of starch accumulation in the two varieties showed a profile of increasing and then decreasing, the expression of starch synthesis-related genes gradually decreased after pollination, demonstrating the complexity of starch synthesis. Future research should focus on construction of a recombinant inbred line population and a mapping population derived from varieties with different starch contents to localize key genes for starch synthesis in sorghum seeds. This study provides a preliminary investigation of the regulatory mechanism of starch synthesis in sorghum grain and lays the foundation for future study of sorghum starch biosynthesis regulatory mechanisms.

## Materials and methods

### Experimental materials and planting methods

The test materials used in this study were sorghum Liaonian3 and Liaoza10. Liaonian3 is a glutinous medium- to late-maturing variety with high starch content and Liaoza10 is a japonica variety. Both varieties were selected by the Institute of Sorghum, Liaoning Academy of Agricultural Sciences. The sorghum seeds used in the study were in full compliance with local and national regulation, and the collection and use of seeds conform to national policies and regulations.The experiment was conducted in May 2020 at the experimental station of Liaoning Academy of Agricultural Sciences (123.54°E, 41.82°N) in a four-row zone with 4 m row length, 0.6 m row spacing, and 20 cm plant spacing, with three independent replicates planted at the same time. Field management was the same as field production.

### Determination of sorghum seed starch content

Amylose content was determined by the dual wavelength method with main and reference wavelengths (λ1 and λ2, respectively) of 620 nm and 479 nm for amylose and 556 nm and 737 nm for amylopectin. The sum of the amylose and amylopectin content was recorded as the total starch content^25^.

### RNA extraction, library construction, and sequencing

The middle cotyledons of the spikes were stored in liquid nitrogen at 3 d, 18 d, and 30 d after pollination and sent to Guangzhou Gene Denovo Biotechnology Co (Guangzhou, China).

Total RNA was extracted using a Trizol kit (Invitrogen, Carlsbad, CA, USA). RNA quality was assessed using an Agilent 2100 Bioanalyzer (Agilent Technologies, Palo Alto, CA, USA) and agarose gel electrophoresis using RNase-free reagent. After extraction of total RNA, eukaryotic mRNA was enriched using Oligo(dT) beads and prokaryotic mRNA was enriched by removal of rRNA with the Ribo-Zero Magnetic Kit (Epicentre, Madison, WI, USA). Enriched mRNA was then fragmented into short pieces using fragmentation buffer and reverse transcribed into cDNA using random primers. The second-strand cDNA was synthesized using DNA polymerase I, RNase H, dNTPs, and buffer. The cDNA fragments were then purified using a QiaQuick PCR extraction kit (Qiagen, Venlo, The Netherlands), the ends were repaired, poly(A)s were added, and the products ligated to Illumina sequencing connectors. Ligated products were selected by size using agarose gel electrophoresis, then amplified with PCR. PCR product was sequenced using an Illumina HiSeq2500 by Gene Denovo Biotechnology Co.

### Data quality control and sequence comparison analysis

Raw reads were quality-controlled using fastp to filter out low-quality data and retain clean reads. Ribosomal RNA sequences were removed using Bowtie2^26^ and compared to the sorghum reference genome (https://genome.jgi.doe.gov/portal/pages/dynamicOrganismDownload.jsf?organism=Sbicolor). Sequences that could not be aligned to the genome were filtered out, then sequencing quality assessment, gene expression, and other analyses were performed^27,28^.

### Differential gene screening

#### Quantification of gene expression

The mapped reads for each sample compared to the reference genome were extracted using StringTie v1.3.1^29,30^. The fragment per kilobase million (FPKM) values of each transcribed region were used to quantify the expression abundance and variation of that gene. FPKM was calculated as follows:

$$ {\text{FPKM}} = \tfrac{{10^{6} {\text{C}}}}{{{\text{NL}}/10^{3} }} $$

where FPKM is the expression level of a gene, C is the number of fragments mapped to the gene, N is the total number of fragments mapped to the reference gene, and L is the number of bases in the gene. Measuring expression levels using FPKM values eliminates the effects of different gene lengths and the amount of sequencing data, so calculated gene expression levels can be directly compared between samples^29,30^.

#### Screening for differentially expressed genes

The genes that were differentially expressed between groups were screened and analyzed using DESeq2. Genes or transcripts with a False Discovery Rate (FDR) below 0.05 and an absolute fold change of ≥ 2 were considered to be differentially expressed^31,32^.

### Profile analysis

Gene expression was analyzed for three time points in each sorghum variety, and profiles in gene expression (patterns) were identified by clustering. From the clustering results, the set of genes that matched certain biological characteristics (e.g., a continuous increase or decrease in expression) were selected. Profile analysis was performed using ShortTime-series Expression Miner software with the following parameters: -pro 20 -ratio 1.0000 [log2(2) = 1,log2(1.5) = 0.5849625,log2(1.2) = 0.2630344]^32^. Gene ontology (GO) and Kyoto Encyclopedia of Genes and Genomes (KEGG) biochemical pathway enrichment analyses were performed for the genes in each profile, and a *p*-value was calculated by hypothesis testing. *p*-values were corrected with FDR^33^, and GO terms and pathways with *q* ≤ 0.05 were defined as significantly enriched in that profile. Functional annotation and GO analyses were performed with Blast2go 4.1^34^, and KEGG Pathway enrichment analyses were performed using KOBAS 2.0 (http://kobas.cbi.pku.edu.cn)^35^.

## Supplementary Information

Supplementary Information 1.

Supplementary Information 2.

Supplementary Information 3.

## Data Availability

The sequencing data is available in NCBI under accession number PRJNA804345. Datebase link: https://www.ncbi.nlm.nih.gov/sra/?term=PRJNA804345.
