# Supplementary material for: Transcriptomic analysis of starch accumulation patterns in different glutinous sorghum seeds
Source: Sci Rep. 2022 Jul 1;12:11133. doi: 10.1038/s41598-022-15394-1 (PMC9249802; doi:10.1038/s41598-022-15394-1)
Supplement: Supplementary file 1 — Supplementary Information 1. [file 41598_2022_15394_MOESM1_ESM.pdf]

Appendix 1 The orthologous relationship of starch-related genes in rice

Gene ID in sorghum	Starch-related gene in rice
entrzID_8075199<br>*entrzID_110436912*<br>*entrzID_8065370*<br>*entrzID_8056206*<br>*entrzID_8078919*<br>*entrzID_8083539*<br>*entrzID_8069792*<br>*entrzID_8072062*	*OsAmy3D*（*RAmy3D*），*RAmy1A*
entrzID_8066807<br>*entrzID_8085013*	*OsSSIIIa*（*Flo5*），*OsSSIIIb*
entrzID_8081295	*ALK*（*OsSSIIa*）
entrzID_110430978	*OsSSI*（*SSS1*）
entrzID_8057926<br>*entrzID_8068390*	*wx*（*qGC-6; Wx-mq; Wx-op*）, *RAG2*
entrzID_8068151	*RSR1*
entrzID_8066439	*OsSSIIb*
entrzID_8059673	*du3*（*OsCBP20*）
entrzID_8059621	*OsBEIIb*
entrzID_8056518	*SSG4*
