# Supplementary figures and images for: Transcriptomic analysis of starch accumulation patterns in different glutinous sorghum seeds
Source: Sci Rep. 2022 Jul 1;12:11133. doi: 10.1038/s41598-022-15394-1 (PMC9249802; doi:10.1038/s41598-022-15394-1)

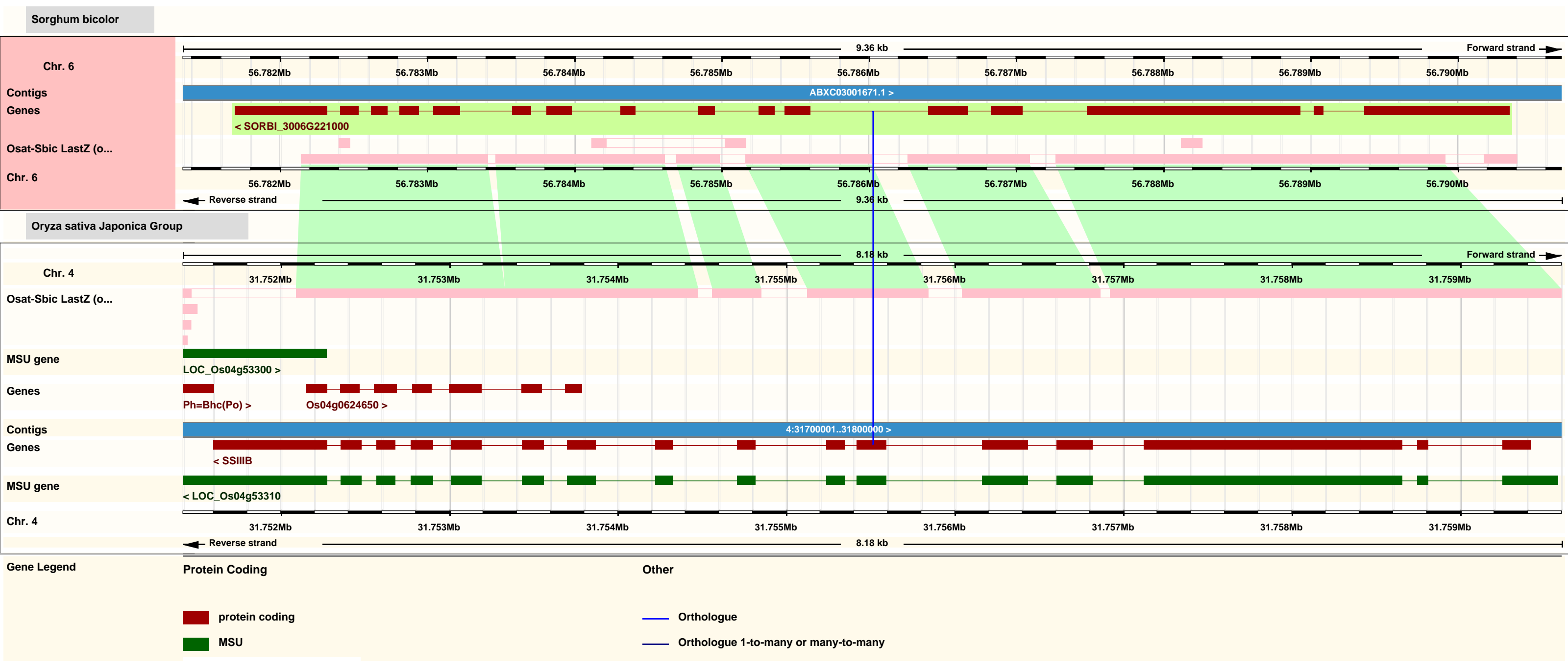

Supplement: Supplementary file 2 — Supplementary Information 2. [file 41598_2022_15394_MOESM2_ESM.pdf]
