# Supplementary material for: Transcriptomic analysis of starch accumulation patterns in different glutinous sorghum seeds
Source: Sci Rep. 2022 Jul 1;12:11133. doi: 10.1038/s41598-022-15394-1 (PMC9249802; doi:10.1038/s41598-022-15394-1)
Supplement: Supplementary file 3 — Supplementary Information 3. [file 41598_2022_15394_MOESM3_ESM.docx]

**Appendix**

**Appendix 1 The orthologous relationship of starch-related genes in rice**

**Appendix 2 The degree of similarity between *entrzID_8066807* and *OsSSIIIa***
